# Editorial: Plant Development: From Cells to Systems Biology

**DOI:** 10.3389/fpls.2021.810071

**Published:** 2021-12-17

**Authors:** Stefan de Folter, Klaus Palme, José Manuel Pérez-Pérez

**Affiliations:** ^1^UGA-LANGEBIO, Centro de Investigación y de Estudios Avanzados del Instituto Politécnico Nacional (CINVESTAV-IPN), Irapuato, Mexico; ^2^Faculty of Biology, Albert-Ludwigs-University of Freiburg, Freiburg, Germany; ^3^Instituto de Bioingeniería, Universidad Miguel Hernández, Elche, Spain

**Keywords:** plant development, meristems, gene networks, plastids, cell cycle, roots

This Research Topic was organized in relation to the two VISCEA conferences (https://viscea.org/), the *Plant Development: Systems Approach II* and *Plant Cells & Tissues in Vitro III*, held in Vienna, Austria, on June 28–29 and July 1-2, 2019, respectively. During four intensive days, the latest research was presented on a broad range of topics related to plant development such as organ development, regeneration, hormone signaling, epigenetics, flowering, and embryo and seed development. The Research Topic had as aim to show a glimpse of the studies or related studies presented on these two conferences, but as well was open for other contributions. Shortly after this Research Topic started, the world was hit by the COVID-19 pandemic, which had, and still in various parts of the world has, a tremendous effect. Related to the Academic setting, many laboratories were locked for several months or even up to more than 1 year. We hope that our colleagues worldwide are doing well, and life is coming back to “normal.” Due to the COVID-19 pandemic, the Research Topic stayed modest in size, though, four excellent contributions were published during 2020 and 2021.

The contributions describe results at the cellular, tissue, and organ level. Plant hormones are crucial for plant growth, development and regeneration, and various presentations were related to hormones during the two congresses. Hormones are sensed at the cellular level by receptor proteins. In this issue, Cerbantez-Bueno et al., studied the expression patterns and function of the three ARABIDOPSIS HISTIDINE KINASE (AHK) cytokinin receptors during gynoecium development in *Arabidopsis thaliana*. Genetic analysis indicated that the three AHK receptors are needed for gynoecium development, and that they have both redundant and specialized functions.

Precise plant cell cycle regulation is important for plant growth. Plants grow due to the coordinated action of mitotic cell cycle and cell expansion. In this issue, Schwedersky et al., focused on the APC/C subunit 11 (APC11), a member of the Anaphase Promoting Complex/Cyclosome (APC/C), which is a conserved E3 ubiquitin ligase complex that targets specific substrates for degradation through the 26S proteasome. Ubiquitylation of major regulators of mitosis triggered by APC/C is required for cell cycle progression. The authors studied two knockdown mutants and an amiRNA mutant of *APC11*, and found that meristematic activity was reduced in shoot and root meristems in these mutants. The authors concluded that APC11 is important for regulating the level of mitotic cyclins in meristem tissues during cell division, through APC/C-mediated ubiquitination, and thereby affecting the overall plant growth and viability.

Protein function is dependent on its specific location within the cell which, in turn, depends on known peptide signals in its sequence. Most plastid proteins are nucleus-encoded and are synthesized as precursors with N-terminal targeting signals, which are called transit peptides (TPs). TPs are necessary for protein import into plastids. In this issue, Eseverri et al., report on the function of three TPs from photosynthesis-related proteins and one TP from a protein involved in ammonium assimilation. Fusion of these Arabidopsis TPs to GFP gave higher efficiency of plastid import in Arabidopsis and rice protoplasts. As the authors mention, these results open the possibility for efficient import of nuclear-encoded proteins into plastids, providing new biotechnological tools for crop improvement strategies.

In root crops (e.g., cassava, sweet potato, carrot, etc.), yield of storage roots is mainly determined by secondary (i.e., radial) growth driven by the vascular cambium. Hoang et al., give an overview of gene regulatory networks (GRNs) that guide storage root development. The authors reanalyze gene expression data generated for major root crops and discuss key conserved GRNs involved in the vascular cambium-driven secondary growth in storage roots using information available in Arabidopsis. They also discussed engineering strategies for some of these GRNs that could improve root crop yields.

This Editorial Note and Research Topic articles report only on a small part of what was presented during the two VISCEA conferences. We very much thank the authors for their contributions and the reviewers for their help. Again, we hope that our colleagues and their families are coming back to a more “normal” life, and that science keeps us all connected.

## Author Contributions

All authors listed have made a substantial, direct, and intellectual contribution to the work and approved it for publication.

## Funding

Research in the JMP-P lab was funded by the Ministerio de Ciencia e Innovación of Spain, grant no. RTI2018-096505-B-I00, and the European Regional Development Fund (ERDF) of the European Commission. Research in the SdF lab is funded by the Consejo Nacional de Ciencia y Tecnología of Mexico (CONACyT) grant CB-2017-2018-A1-S-1012. SdF also acknowledges the Marcos Moshinsky Foundation and the H2020-MSCA-RISE 2020 EVOfruland project 101007738.

## Conflict of Interest

The authors declare that the research was conducted in the absence of any commercial or financial relationships that could be construed as a potential conflict of interest.

## Publisher's Note

All claims expressed in this article are solely those of the authors and do not necessarily represent those of their affiliated organizations, or those of the publisher, the editors and the reviewers. Any product that may be evaluated in this article, or claim that may be made by its manufacturer, is not guaranteed or endorsed by the publisher.

